# Proteinase PrtP impairs lactococcin LcnB activity in *Lactococcus lactis* BGMN1-501: new insights into bacteriocin regulation

**DOI:** 10.3389/fmicb.2015.00092

**Published:** 2015-02-10

**Authors:** Goran Vukotic, Nemanja Mirkovic, Branko Jovcic, Marija Miljkovic, Ivana Strahinic, Djordje Fira, Zorica Radulovic, Milan Kojic

**Affiliations:** ^1^Laboratory for Molecular Microbiology, Institute of Molecular Genetics and Genetic Engineering, University of BelgradeBelgrade, Serbia; ^2^Chair of Biochemistry and Molecular Biology, Faculty of Biology, University of BelgradeBelgrade, Serbia; ^3^Department for Food Microbiology, Faculty of Agriculture, University of BelgradeBelgrade, Serbia

**Keywords:** lactococci, bacteriocin activity, LcnB, proteinase PrtP, digestion, medium dependent activity

## Abstract

Proteinases and bacteriocins are of great importance to the dairy industry, but their interactions have not been studied so far. *Lactococcus lactis* subsp. *lactis* BGMN1-5 is a natural isolate from homemade semi-hard cheese which produces two bacteriocins (Lactococcin B and LsbB), as well as proteinase PrtP. A medium-dependent increase in the bacteriocin LcnB activity of *L. lactis* BGMN1-501, a derivate of *L. lactis* subsp. *lactis* BGMN1-5, was shown to be accompanied by a decrease in its promoter activity. A similar effect of media components on gene expression was reported for proteinase PrtP, whose gene is co-localized on the same plasmid as the *lcnB* gene. Thus, the PrtP-LcnB interplay was investigated. Single gene knockout mutants were constructed with disrupted *prtP* or *lcnB* genes. PrtP^-^ mutants showed higher bacteriocin activity that had lost its growth medium dependence, which was in contrast to the original strain. When LcnB from this mutant was combined with proteinase from the LcnB^-^ mutant *in vitro*, its activity was rendered to the original level, suggesting that proteinase reduces bacteriocin activity. We propose a new model of medium dependent expression of these genes with regard to the effects of their interaction *in vivo*.

## INTRODUCTION

*Lactococcus lactis* is the best characterized species of lactic acid bacteria (LAB) and one of the most studied and utilized Gram-positive bacteria. It is used as a starter culture for manufacturing a wide variety of fermented milk products, either as a single culture or in a mixture with other LAB. It’s most important trait is the production of lactic acid and the acidification of milk, which is used in numerous ways in the production of fermented milk products. It leads to curdling of milk and at the same time prevents the growth of undesirable bacteria. Among other prominent and well-known features of lactococci is the production of proteolytic enzymes and a diverse array of bacteriocins (Lahtinen et al., 2012).

Proteinases, as part of a complex proteolytic system, confer on lactococci the ability to multiply quickly in milk, which is of great importance for the dairy industry. Furthermore, proteinases are involved in the development of organoleptic properties in fermented milk products and their activity may affect consumers’ health, especially regarding the activity of peptides, which they release from milk proteins. Diverse bioactive peptides liberated by milk proteolysis have been described as having immunomodulatory, antioxidative, antihypertensive, and antimicrobial properties (Hayes et al., 2007). The occurrence and composition of bioactive peptides depend mainly on the substrate specificity of proteinases applied in food processing.

Numerous lactococci produce bacteriocins, ribosomally synthesized antimicrobial peptides, which can vary widely in structure and mode of action. This diversity has led to the division of bacteriocins into several classes with multiple subclasses (Cotter, 2014). Bacteriocins are very important for colonization and survival in niches such as milk or cheese. In such highly competitive conditions they provide an advantage over closely related species, which are generally their main targets. Nevertheless, some bacteriocins are active against a wide variety of pathogens, either Gram-positive or Gram-negative, which is why they have been widely applied in food preservation and are very important in the search for new antibiotics. Besides these bactericidal effects, bacteriocins can also serve as signaling molecules, enabling bacterial communication, and coordination through quorum sensing. In addition, this bacteriocin-driven communication can also take place between different bacterial species (Calasso et al., 2013), as well as between host immune cells and colonizing bacteria (Meijerink and Wells, 2010; van Hemert et al., 2010).

*Lactococcus lactis* subsp. *lactis* BGMN1-5 is a natural isolate from homemade semi-hard cheese which produces two potent bacteriocins [Lactococcin B (LcnB) and LsbB] and proteinase PrtP (Gajic et al., 1999). Its derivate, *L. lactis* subsp. *lactis* BGMN1-501, obtained by plasmid curing, retained genes for LcnB and proteinase PrtP, which colocalize on the same plasmid, pMN80 (Kojic et al., 2006). PrtP is a large (~200 kDa) extracellular enzyme that is attached to the cell wall at its C terminus, although autocatalytically truncated active molecules are abundant in the extracellular environment (Laan and Konings, 1989). LcnB is a small (~3 kDa), hydrophobic, positively charged peptide with an antimicrobial spectrum limited to *Lactococcus* species (Venema et al., 1997). Its mode of action relies on binding to components of the mannose phosphotransferase system (man-PTS) of susceptible cells, causing leakage of cellular components across the membrane (Diep et al., 2007).

It has been observed previously that the activity of LcnB is affected by media peptide concentration. Peptide-rich media were proven to promote activity, while peptide-poor media minimize LcnB activity. This was reported in experiments with both chemically defined media (Gajic et al., 1999; Miladinov et al., 2001), and GM17 media (Venema et al., 1997)

In this research we focus on the interaction between proteinase PrtP and bacteriocin LcnB in BGMN1-501, given that, so far, no information exists on this matter. We explored the promoter activity of *lcnB* during growth in different casitone concentrations, and showed that expression of LcnB is silenced in a peptide rich environment, which is contrary to the prevailing belief. Moreover, we offer evidence of the digestion of LcnB by proteinase PrtP, which drastically, but not completely, reduces its activity. Bringing these facts together, we propose a model of *lcnB* regulation which sheds new light on the complex interaction between these proteins and their environment.

## MATERIALS AND METHODS

### BACTERIAL STRAINS AND PLASMIDS

The bacterial strains and plasmids used in this study are listed in **Tables 1** and **2**. Lactococcal strains were grown in M17 medium (Merck) supplemented with D-glucose (0.5% w/v; GM17) at 30°C, unless otherwise noted. *Escherichia coli* DH5α and EC101, used for cloning and propagation of constructs, were grown in Lauria-Bertani (LB) broth (Miller, 1972) aerobically at 37°C. Agar plates were made by adding 1.5% (w/v) agar (Torlak Belgrade, Serbia) to the liquid media. Transformants of lactococci were selected on GM17 plates containing 10 μg/ml of erythromycin (Sigma–Aldrich Chemie GmbH, Deisenhofen, Germany) or 7.5 μg/ml of chloramphenicol in the final concentration. *E. coli* transformants were selected on LB plates containing 300 μg/ml of erythromycin, 35 μg/ml of chloramphenicol, or 100 μg/ml of ampicillin, depending on the plasmids used. When necessary, 5-bromo-4-chloro-3-indolyl-D-galactoside (X-gal; Fermentas, Vilnius, Lithuania) was added to LB or GM17 medium plates at a final concentration of 50 μg/ml for blue/white screening of colonies carrying vectors with clones.

**Table 1:** Bacterial strains used in this study.

Strains	Relevant characteristic(s)	Source or reference
***Lactococcus lactis* subsp. *lactis***
BGMN1-501	Derivate of BGMN1-5 with pMN80 plasmid, LcnB^+^, PrtP^+^, LcnB^r^	Kojic et al. (2006)
BGMN1-596	Plasmid free derivate of BGMN1-5, LcnB^-^, PrtP^-^, LcnB^s^	Kojic et al. (2006)
BGMN1-501/pG^+^host9prtP	PrtP^-^, LcnB^+^, LcnB^r^	This work
BGMN1-501/pG^+^host9lcnB	PrtP^+^, LcnB^-^, LcnB^r^	This work
***L. lactis* subsp. *cremoris***
NCDO712	PrtP^+^, Lac^+^	Gasson (1983)
MG7284	Plasmid free derivative of NCDO712, PrtP^-^, Lac^-^	Gasson (1983)
MG7284/pNZ8150lacZ1PlcnB		This work
MG7284/pNZ8150lacZ1		This work
***Escherichia coli***
DH5α	Λ^-^ Φ*80dlacZ*ΔM15*Δ(lacZYA-argF)U169 recA1 endA1 hsdR17(rk^-^mk^-^) supE44thi-1gyrA relA1*	Hanahan (1983)
EC101	JM101 containing *repA* gene of pWV01 in chromosome	Law et al. (1995)
DH5α/pGem-T-easylcnB		This work
EC101/pG^+^host9prtP		This work
EC101/pG^+^host9lcnB		This work

**Table 2:** Plasmids used in this study.

Plasmids	Relevant characteristic(s)	Source or reference
pG^+^host9	Em^r^, thermosensitive vector	Maguin et al. (1996)
pG^+^host9prtP	pG^+^host9 carrying fragment of *prtP* gene	This work
pG^+^host9lcnB	pG^+^host9 carrying whole *lcnB* gene	This work
Q6	M13mp10 with 1.1 kbp carrying fragment of prtP gene	Kojic et al. (1991)
pUT/Km	Mini-Tn*5lacZ1*	de Lorenzo et al. (1990)
pBSlacZ1		This work
pNZ8150	*Sca*I site used for translational fusions, standard vector; Cm^r^	Mierau and Kleerebezem (2005)
pNZ8150lacZ1		This work
pNZ8150lacZ1PlcnB		This work
pGem-T-Easy	3015 bp; Amp^r^; PCR cloning vector	Promega
pGem-T-easylcnB	pGEM-T-easy carrying *lcnB* gene	This work
pGEM-T-easyP*_lcnB_*	pGEM-T-easy carrying *lcnB* promoter	This work
pBlueScript	2961 bp; Amp^r^; PCR cloning vector	Stratagene

### BACTERIOCIN ACTIVITY ASSAY

The bacteriocin activity of *L. lactis* subsp. *lactis* BGMN1-501, recombinant strains and mutants was evaluated by an agar-well diffusion test (Lozo et al., 2004). Tested strains were grown in GM17 medium with different concentrations of casitone (0.5%, 1%, 2%, 4%, 6%, and 8%; Difco Laboratories, Becton Dickinson and Company, Franklin Lakes, NJ, USA). The cell-free supernatants of tested strains were obtained by centrifugation (13000 rpm for 10 min) of 16-h overnight culture and subsequent filtration through a 0.45 μm filter to obtain LcnB extracts. *L. lactis* subsp. *lactis* BGMN1-596 was used as an indicator strain. Clear zones of inhibition around the wells were used as positive signals for bacteriocin production.

### PROTEINASE ACTIVITY ASSAY

For proteinase activity analysis of the obtained mutants, the method described by Kojic et al. (1991) was used. Cells were induced by growing on milk-citrate-agar (MCA) plates [containing 4.4% reconstituted milk, 0.8% Na-citrate, 0.1% yeast extract, 0.5% glucose, and 1.5% agar (w/v)] for 48 h at 30°C prior to collection. Collected fresh cells were resuspended in 0.1 mol/L sodium phosphate buffer (pH 7). β-casein (Sigma–Aldrich) was dissolved in the same buffer at 5 mg/ml final concentration. The cell suspension containing 10^9^ cells per mL was mixed with β-casein solution at a 1:1 (v/v) ratio. After incubation for 4 h at 30°C, the cells were pelleted by centrifugation (13000 ×*g*, 10 min), the clear supernatant fluid was taken off and samples were prepared for SDS-PAGE.

For *in vitro* testing of the proteinase’s effect on bacteriocin activity, cell-free proteinase extract was prepared. Cells were grown on MCA plates, collected and re-suspended in 0.1 mol/L sodium phosphate buffer (pH 7), incubated at room temperature for 30 min, pelleted by centrifugation and supernatants were collected prior to reaction.

Samples for SDS-PAGE were mixed with sample loading buffer (0.125 mol/L TrisHCl, pH 6.8, 0.01 mmol/L EDTA, 4% SDS, 25% glycerol, 5% 2-mercaptoethanol, and 0.07% bromophenol blue) at a 1:1 ratio by volume. Before loading, samples were heated at 100°C for 5 min.

### INTEGRATION OF PLASMID pG^+^HOST9 INTO pMN80

The plasmid pG^+^host9, a thermosensitive erythromycin-resistant derivate of the plasmid pWVO1 (Maguin et al., 1992), was used to construct the integrative vector for disrupting *prtP* and *lcnB* genes, located on plasmid pMN80 of *L. lactis* BGMN1-501. A 1.1 kbp fragment containing the DNA region coding for the active site of PrtP proteinase of *L. lactis* subsp. *cremoris* Wg2, necessary for inactivation based on homologous recombination, was obtained from M13-Q6 vector (Kojic et al., 1991) using *Eco*RI and *Hin*dIII restriction enzymes and then was cloned into pG^+^host9 to yield pG^+^host9prtP. A PCR fragment of 578 bp containing gene *lcnB* was cloned into commercial pGem-T-easy vector (Promega, Madison, WIS, USA) to yield pGem-T-easylcnB, and subsequently cloned into pG^+^host9 vector digested with *Eco*RI, resulting in the construct pG^+^host9lcnB. Constructs pG^+^host9prtP and pG^+^host9lcnB were used for transformation of BGMN1-501. Transformants obtained at 28°C were streaked onto fresh GM17 agar plates containing erythromycin (10 μg/ml) and incubated at 37°C for 48 h to enable integration.

### MOLECULAR TECHNIQUES

Pulsed-field gel electrophoresis (PFGE) was used for clonal confirmation, as described by Kojic et al. (2005). Total DNA from lactococci was isolated by the modified methods described by Hopwood et al. (1985). The method used for mini-prep isolation of plasmid DNA from lactococci was described by O’sullivan and Klaenhammer (1993). For isolation of plasmid DNA from *E. coli*, a QIAprep Spin Miniprep Kit was used according to the manufacturer’s recommendations (Qiagen, Hilden, Germany). All digestions with restriction enzymes were conducted according to the supplier’s instructions (Fermentas, Lithuania). T4 DNA ligase (Agilent Technologies, Santa Clara, CA, USA) was used for DNA ligation, according to the manufacturer’s recommendation. Plasmid constructs were introduced into lactococci by electroporation using an Eporator (Eppendorf, Hamburg, Germany) using the methodology described by Holo and Nes (1989). The sets of specific primers used in this study are listed in **Table 3**. KapaTaq DNA polymerase (KapaBiosystem, Inc., Boston, MA, USA) was used to amplify DNA fragments by PCR using a GeneAmp PCR system 2700 thermal cycler (Applied Biosystems, Foster City, CA, USA). DNA fragments were purified from agarose gel using a QIAquick Gel extraction kit as described by the manufacturer (Qiagen). PCR products were purified with a QiaQuick PCR purification kit (Qiagen) according to the protocol of the supplier. Obtained and purified PCR products were sequenced by the Macrogen Sequencing Service (Macrogen, Netherlands) and analyzed by using the BLAST algorithm. Commercial p-Gem-T-Easy (Promega) vector was used for cloning PCR products.

**Table 3:** Sequence of specific primers used in this study.

Primer name	Sequence of primer	Template	Source or reference
LactABM-F	5′-gaagaggcaatcagtagag- 3′	pMN80 plasmid DNA	Alegría et al. (2010)
LactB-R	5′-ccaggattttctttgatttacttc- 3′	pMN80 plasmid DNA	Alegría et al. (2010)
PlcnB-FW	5′-ctgcagagttattaacatttgttaacg- 3′	pMN80 plasmid DNA	This work
PlcnB-REV	5′-gagctcgatttttcataataatctcc- 3′	pMN80 plasmid DNA	This work

### PLASMID CONSTRUCTION

A transcription fusion vector for lactococci (named pNZ8150lacZ1) was constructed as follows. The *lacZ1* gene without promoter was removed from pUT/Km vector using *Eco*RI-*Hin*dIII restriction enzymes and cloned first into pBlueScript vector digested with the same enzymes, yielding the construct pBSlacZ1. From pBSlacZ1, the *lacZ1* gene was re-cloned into pNZ8150 vector (Mierau and Kleerebezem, 2005) using *Pst*I-*Hin*dIII restriction enzymes, resulting in construct pNZ8150lacZ1. This vector contained restriction enzyme sites for cloning promoter fragments upstream of the *lacZ1* gene (*Sca*I, *Pst*I, *Eco*RI, *Sma*I, and *Bam*HI). The orientation and position of the *lacZ1* gene in pNZ8150lacZ1 was confirmed by sequencing in both orientations. The *lcnB* promoter region (P*_lcnB_*) was amplified using specific primers, PlcnB-FW and PlcnB-REV (**Table 3**), and cloned into pGEM-T-easy, yielding pGEM-T-easyPlcnB. The promoter region was removed from pGEM-T-easyPlcnB by *Pst*I and *Eco*RI restriction enzymes and cloned into pNZ8150lacZ1 vector digested with the same enzymes, to yield construct pNZ8150lacZ1PlcnB. The activity of *lcnB* promoter in pNZ8150lacZ1PlcnB was confirmed by a β-galactosidase activity assay of *L. lactis* subsp. *cremoris* MG7284 carrying pNZ8150lacZ1PlcnB (P*_lcnB_* transcription fusion) and pNZ8150lacZ1 (negative control).

### β-GALACTOSIDASE ACTIVITY ASSAY

The activity of β-galactosidase was determined by assaying the degradation of ortho-nitrophenyl-β-D-galactopyranoside (ONPG; Sigma–Aldrich) at 30°C using a modification of the method described by Miller (1972). Lactococcal cells from the logarithmic phase were harvested by centrifugation and resuspended in 500 μl of PP buffer containing 4 mg of lysozyme per milliliter. Degradation of the cell wall was conducted for 30 min at 37°C. After that, cells were harvested by centrifugation (5 min at 5000 ×*g*). Cell pellets were resuspended in 500 μl of Z-buffer and the protocol was continued as described previously (Miller, 1972).

### *IN VITRO* TESTING OF EFFECT OF PROTEINASE EXTRACT ON LcnB ACTIVITY

LcnB extracts from both BGMN1-501 and BGMN1-501/pG^+^host9prtP were incubated with proteinase extract from BGMN1-501/pG^+^host9lcnB (LcnB^-^, PrtP^+^). The extracts were mixed at a 1:1 ratio, and incubated at 30°C for 120 min. In parallel, LcnB extracts from both strains were incubated with NaPi buffer (0.1 mol/L, pH 7) at a 1:1 ratio at 37°C for 120 min, as a control. Proteinase PrtP’s effect on LcnB activity was tested in the same conditions by using proteinase extract isolated from *L. lactis* subsp. *cremoris* NCDO712.

### MEASUREMENT OF LACTOCOCCIN B GROWTH INHIBITION ZONES’ DIAMETERS

Diameters of growth inhibition zones formed in bacteriocin activity assay from extracts of both BGMN1-501 and BGMN1-501/pG^+^host9prtP were measured using AutoCAD 12 (Autodesk, San Rafael, CA, USA).

## RESULTS

### CONSTRUCTION OF MUTANT STRAINS

**FIGURE 1:**
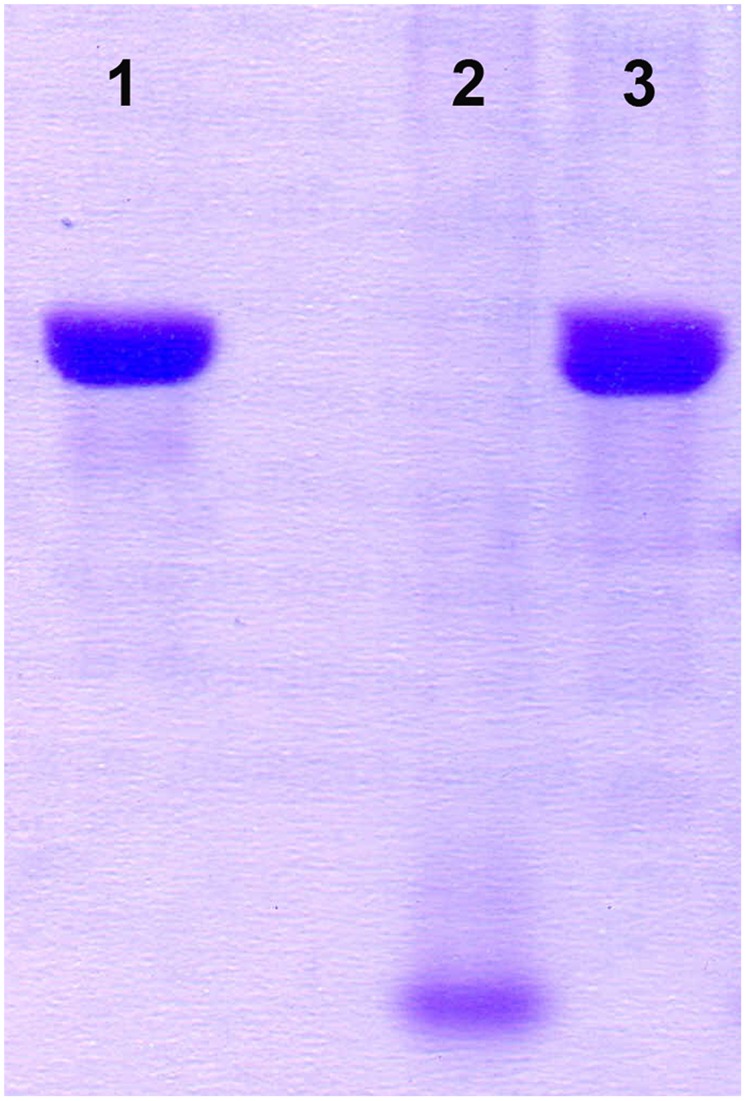
**Analysis of proteinase inactivity of BGMN1-501/pG^+^host9prtP integrant.** (1) β-casein, (2) BGMN1-501, (3) *prtP* integrant.

**FIGURE 2:**
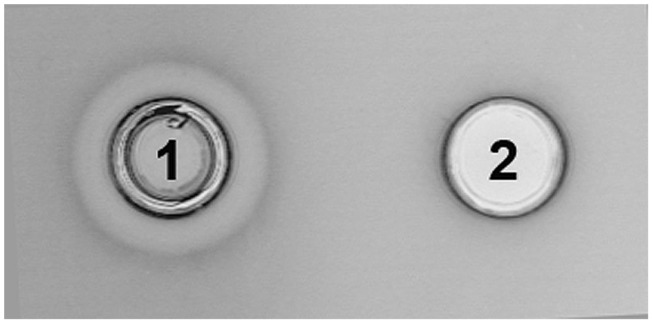
**Analysis of bacteriocin inactivity of BGMN1-501/pG^+^host9lcnB integrant.** (1) BGMN1-501, (2) *lcnB* integrant.

**FIGURE 3:**
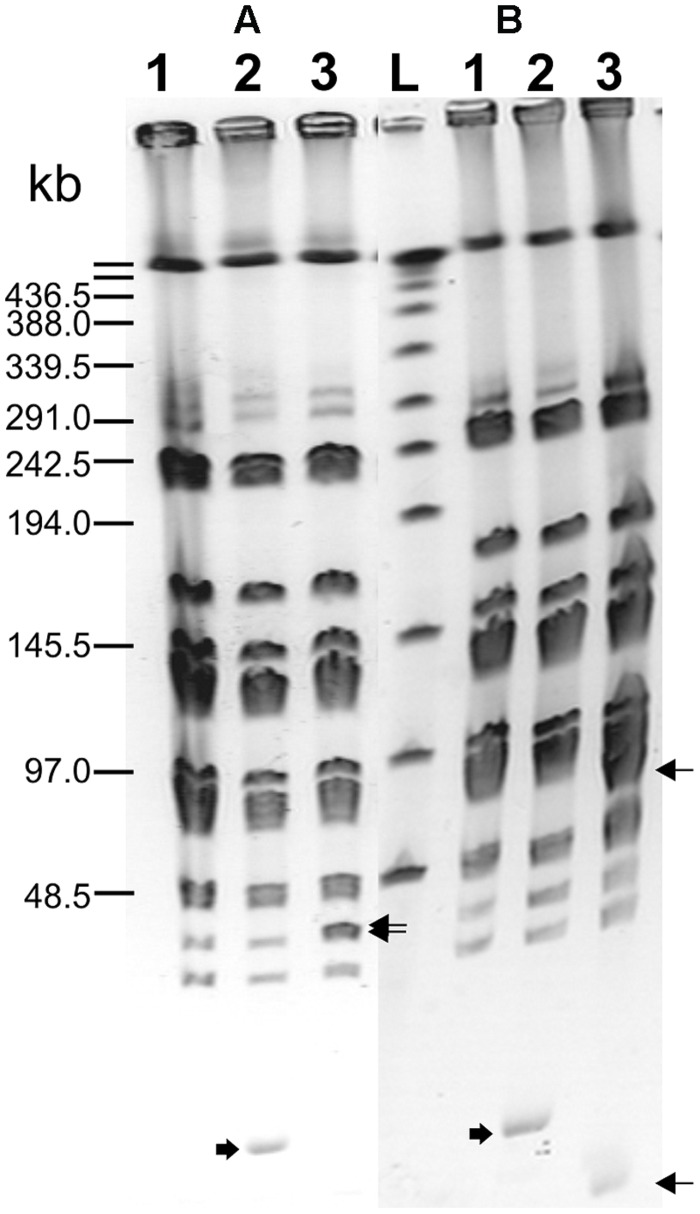
**Pulsed-field gel electrophoresis (*Sma*I macroresriction pattern) of PrtP^**–**^**(A)** and LcnB^**–**^**(B)** mutants of BGMN1-501.** (1) BGMN1-501; (2) BGMN1-501 carrying adequate free pG^+^host with fragment for homologous recombination (growth at 28°C); (3) mutants of BGMN1-501; L, phage λ DNA concatemers. Wide arrows indicate free pG^+^host with a fragment for homologous recombination, sharp arrows indicate the appearance of new *Sma*I fragments in generated mutants after insertion of pG^+^host vectors (pG^+^host introduces additional *Sma*I site).

Integration of the appropriate plasmid constructs (pG^+^host9prtP and pG^+^host9lcnB) into corresponding *prtP* and *lcnB* genes on plasmid pMN80, after growing *L. lactis* subsp. *lactis* BGMN1-501 transformants at 37°C for 48 h, was confirmed by their PrtP^-^ and LcnB^-^ phenotype, as well as PFGE analysis. As expected, the mutants with integrated pG^+^host9prtP construct in the *prtP* gene lost the ability to degrade β-casein (PrtP^-^ mutant; **Figure 1**) and mutants with pG^+^host9lcnB construct integrated into *lcnB* showed LcnB^-^ phenotype (**Figure 2**). PFGE (*Sma*I macrorestriction) analysis of BGMN1-501, transformants with free constructs (pG^+^host9prtP and pG^+^host9lcnB) and mutants confirmed the integration of constructs into plasmid pMN80 at different positions (it is possible to conclude that the distance between *prtP* and *lcnB* on plasmid pMN80 is at least 35 kb; **Figure 3**). Construction of an LcnB^-^ mutant was undertaken in order to eliminate the possible presence of LcnB molecules in proteinase extracts used for *in vitro* LcnB activity analyses.

### BACTERIOCIN ACTIVITY IS MEDIUM DEPENDENT IN BGMN1-501, BUT NOT IN BGMN1-501/pG^+^HOST9prtP (ITS PrtP^-^ MUTANT)

**FIGURE 4:**
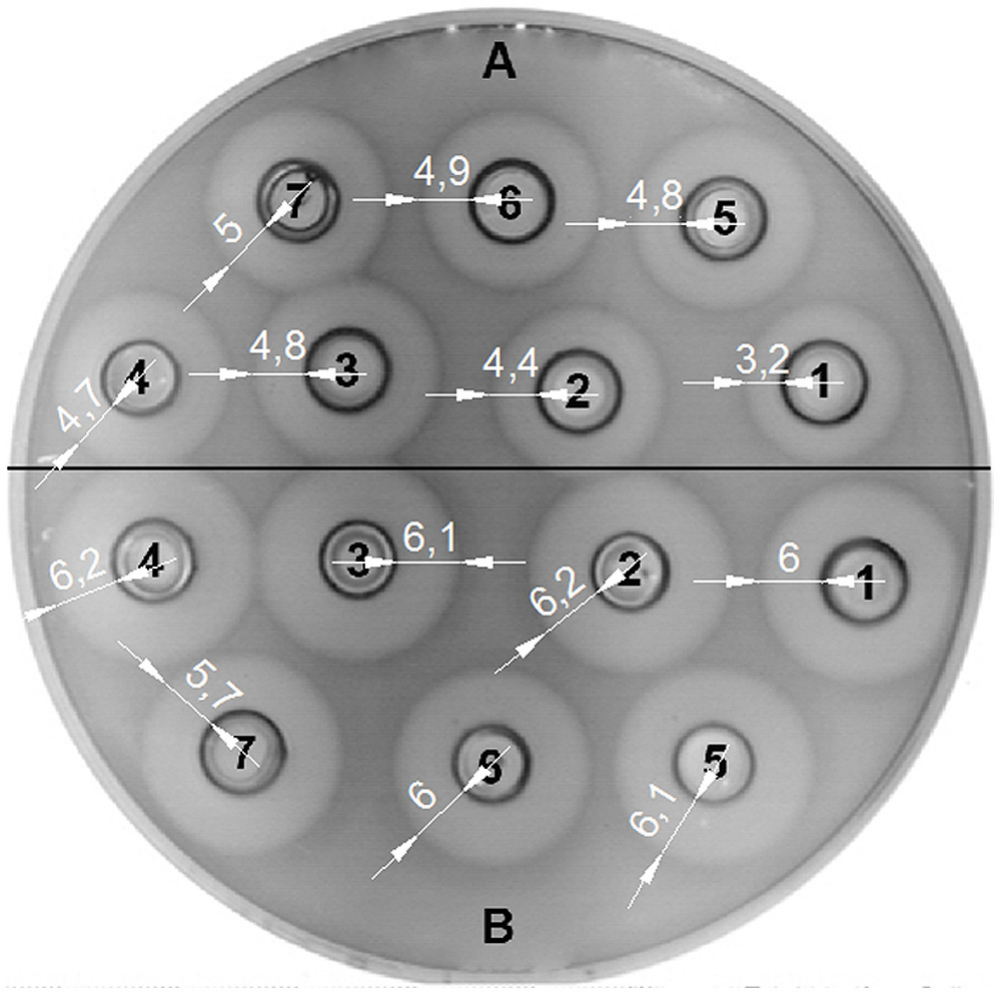
**Activity of LcnB synthesized by BGMN1-501 and BGMN1-501/pG^+^host9prtP (PrtP^–^ mutant) after growth in GM17 media with different concentration of casitone. (A)** LcnB activity of BGMN1-501 after growth in GM17 with (1) 0%; (2) 0.5%; (3) 1%; (4) 2%; (5) 4%; (6) 6%; (7) 8%; of casitone. The zones of growth inhibition enlarge as the concentration of casitone grows;** (B)** LcnB activity of BGMN1-501/pG+host9prtP (PrtP^-^ mutant) after growth in GM17 with (1) 0%; (2) 0.5%; (3) 1%; (4) 2%; (5) 4%; (6) 6%; (7) 8%; of casitone. Note that the zones of growth inhibition are not dependent on casitone concentration in the media.

The bacteriocin activity of BGMN1-501 and its PrtP^-^ mutant was analyzed after 16 h of growth in GM17 with different concentrations of casitone (0% w/v, 0.5% w/v, 1% w/v, 2% w/v, 4% w/v, 6% w/v, and 8% w/v), assayed by the agar well diffusion test on indicator strain BGMN1-596. Casitone is a pancreatic digest of casein mainly consisting of small peptides and amino acids in a ratio of about 4:1 (Marugg et al., 1995). Increasing casitone concentration resulted in higher bacteriocin activity of BGMN1-501 (**Figure 4**). On the contrary, the bacteriocin activity of PrtP^-^ mutant remained unaffected by changes in casitone concentration. It is noteworthy that the zones of inhibition for the mutant were larger than the largest zone BGMN1-501 produced.

### *lcnB* PROMOTER IS UNDER MEDIUM DEPENDENT REGULATION

The transcription fusion vector described here, pNZ8150lacZ1, is the first such vector for expression analysis constructed for lactococci. It has several applicable advantages: favorable selection, multiple restriction enzyme sites, high copy-number, and a common reporter gene. It proved to be reliable, easy to manipulate, and showed highly reproducible results.

**FIGURE 5:**
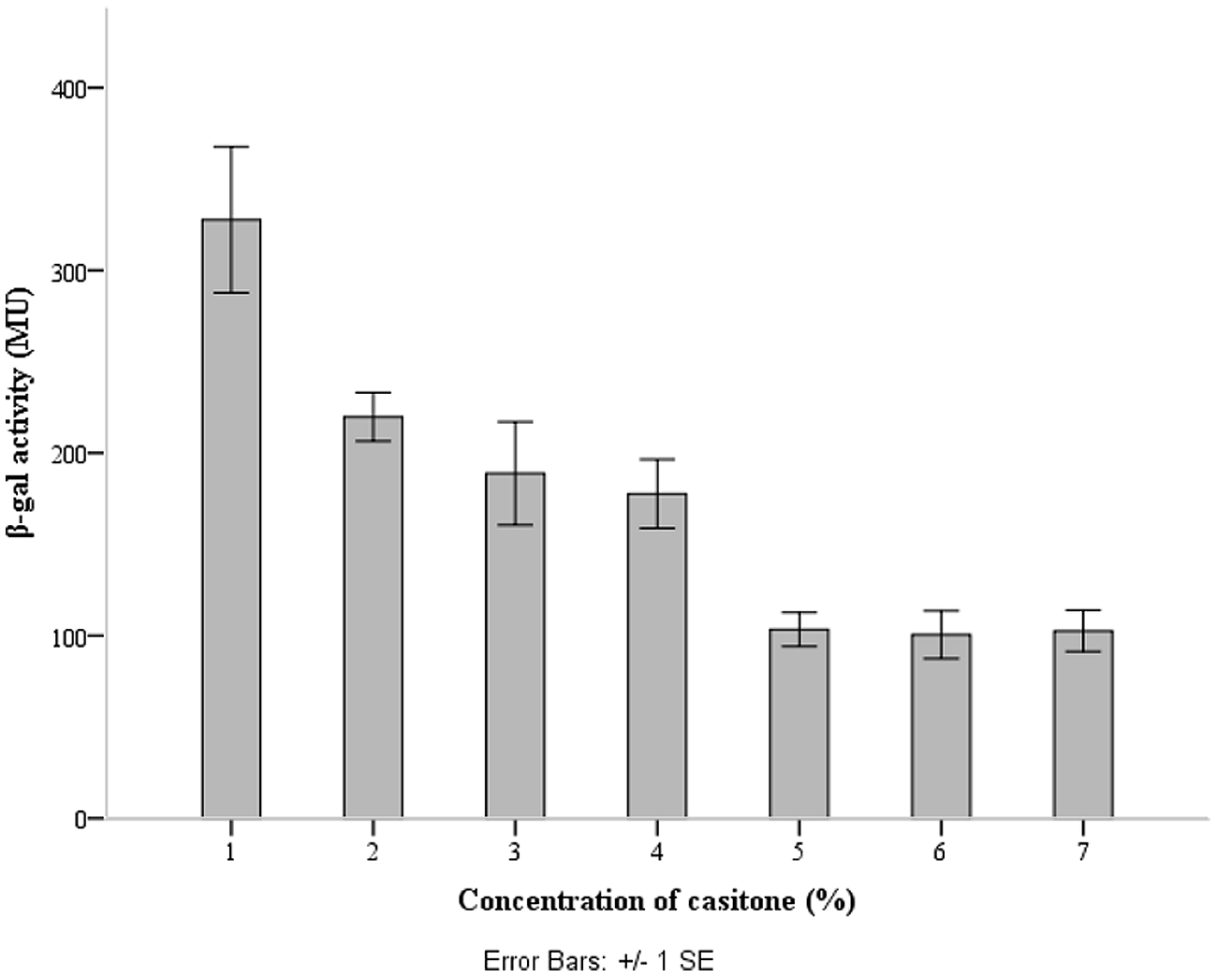
***lcnB* promoter activity measured by β-galactosidase activity assay.** When the cells grow in GM17 medium with a low concentration of casitone, β-galactosidase activity is approximately three times higher than in GM17 medium with a higher concentration of casitone.

The *lcnB* promoter region was cloned upstream of promoter-less *lacZ1* gene in pNZ8150lacZ1 vector, to yield pNZ8150lacZ1PlcnB. The construct was introduced into the proteinase-deficient *L. lactis* subsp. *cremoris* MG7284. The transformant MG7284/pNZ8150lacZ1PlcnB was streaked onto GM17 Petri dishes containing 7.5 μg/ml of chloramphenicol and 50 μg/ml of X-gal, turning apparently blue (data not shown). The β-galactosidase production of transformant MG7284/pNZ8150lacZ1PlcnB was analyzed during growth in GM17 media with different concentrations of casitone (0%, 0.5%, 1%, 2%, 4%, 6%, and 8%). The promoter showed a significant decline in activity in media with higher casitone concentration, but only to a certain point. After 4%, the rise of casitone concentration in the medium had no silencing effect on the *lcnB* promoter (**Figure 5**).

### ACTIVITY OF LACTOCOCCIN B IS REDUCED BY PrtP

**FIGURE 6:**
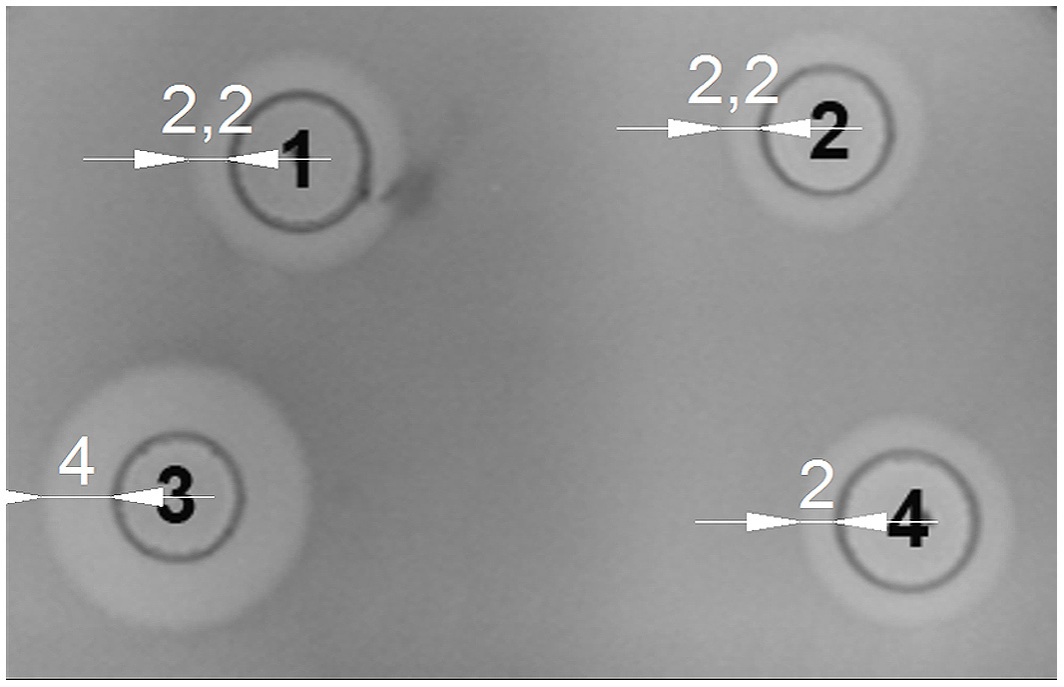
**LcnB activity of BGMN1-501 and its PrtP^**–**^ mutant before and after incubation with PrtP proteinase extract of BGMN1-501/pG^**+**^host9lcnB (LcnB^**–**^ mutant).** (1) LcnB of BGMN1-501 with NaPi; (2) LcnB of BGMN1-501 with PrtP of BGMN1-501/pG^+^host9lcnB; (3) LcnB of PrtP^-^ mutant with NaPi; (4) LcnB of BGMN1-501/pG^+^host9prtP with PrtP proteinase of BGMN1-501/pG^+^host9lcnB. Note that the zone of growth inhibition is rendered to that of the original strain.

We examined whether the activity of LcnB can be modified by PrtP proteinase *in vitro.* LcnB extracts, both from BGMN1-501 and its PrtP^-^ mutant, were mixed in parallel with NaPi buffer and proteinase extract and incubated for 120 min at 30°C. After incubation with proteinase, the LcnB activity of BGMN1-501 was not altered (**Figure 6**, wells 1 and 2). In contrast, the bacteriocin activity of the PrtP^-^ mutant decreased drastically (**Figure 6**, wells 3 and 4). The decrease in activity was monitored at several time points and it was continuous until 1 h of incubation (data not shown). After 1 h, zones of growth inhibition were constant regardless of incubation time with proteinase extract, indicating that in 1 h all of the LcnB was processed by proteinase extract. The same result was observed when proteinase extract from *L. lactis* subsp. *cremoris* NCDO712 (a producer of the same type of PrtP proteinase as BGMN1-501) was used (data not shown).

## DISCUSSION

The production of bacteriocin LcnB, it’s genetics, spectrum of activity, mode of action, and medium dependent activity have been studied in detail (van Belkum et al., 1991; Venema et al., 1993,^1996^; Gajic et al., 1999; Diep et al., 2007). In addition, it was determined that LcnB is sensitive to degradation by several proteases (pepsin, trypsin, chymotrypsin, pronase E, proteinase K) and that high concentrations of peptides in growth media induce its activity. Nevertheless, neither regulation of *lcnB* gene expression, nor the active/binding domain was described.

We analyzed the activity of the *lcnB* gene promoter by cloning it in pNZ8150lacZ1 transcription fusion vector, which we developed for expression analyses in lactococci. For this, we chose pNZ8150 cloning and expression vector with broad range replication and a chloramphenicol resistance marker because it could be cotransformed with other cloning vectors for lactococci, e.g., pAZIL (Kojic et al., 2011). This also enables analysis of the relationship between the promoter region and certain cloned gene(s) in homologous and heterologous hosts. In addition we established and standardized a simple protocol for detection and measurement of β-galactosidase activity in lactococci. Using this system we obtained very reproducible results and high values of β-galactosidase activity in lactococci, in contrast to other authors (Roces et al., 2012). This enabled us to analyze the medium-dependent activity of *lcnB* gene promoter. Through the activity of a common reporter gene (*lacZ*) we were able to quantify promoter activity in different growth media. Surprisingly, it turned out that promoter activity was continuously attenuated, inversely to the increase of casitone concentration. This was completely contrary to the assumptions of previous researchers (i.e., Venema et al., 1997) and from our experiments, which clearly indicated an increase of bacteriocin activity in peptide-rich media (**Figure 4**). It seems that with higher peptide concentration in the growth medium, less of the bacteriocin gene is transcribed, but somehow its activity becomes higher. We assumed that the observed bacteriocin activity indicated the activity of bacteriocin molecules themselves, rather than their number. This activity is apparently changeable *in vivo*, and we suppose that either some kind of enhanced translation or post-translational modification, which is related to different peptide concentrations in media, occurs to bacteriocin. The effect of this modification is so strong that it not only compensates for the fall in bacteriocin expression, but overcomes this effect, making even larger zones of growth inhibition of indicator strains.

Since it has long been known that PrtP expression is highly dependent on casitone concentration in media, the possible impact of proteinase on bacteriocin activity was investigated. *prtP* was knocked out from *L. lactis* BGMN1-501, and mutants were screened for bacteriocin activity in media with different casitone concentrations. Two results were of great importance: (i) zones of growth inhibition were greatly enlarged, suggesting that the lack of PrtP stimulates LcnB activity and (ii) interdependence of LcnB activity and casitone concentration was lost. Zones of growth inhibition were approximately the same size, although cultures were grown in different media. We reasoned that LcnB may be a substrate for PrtP proteinase, given its proteinaceous nature. Indeed, *in vitro* mixing of LcnB and PrtP extracts indicated that proteinase impairs the function of LcnB. After treatment with PrtP, the activity of the mutant’s LcnB was greatly reduced and corresponded perfectly to the activity of the original strain’s LcnB. Moreover, the activity of LcnB extracted from the original strain was not changed with proteinase treatment. We assume that all molecules of LcnB in the original strain had already been digested with intrinsic proteinase, which left no target bonds for additionally added PrtP to cleave.

Lasta et al. (2008) showed that the first six amino acids from the N terminus are crucial for LcnB bactericidal activity, which is why we suppose that cleavage occurs at this location. This assumption is corroborated by the findings of Juillard et al. (1995) who showed that PrtP cleaves, among others, the peptide bond between leucine and glutamine in β-casein. These are the second and third amino acids on the N terminus of bacteriocin LcnB.

We propose a model of *lcnB* expression regulation in which the absence of PrtP in the environment leads to the presence of undigested and hence fully active molecules of LcnB in the media. This happens in a peptide rich environment where growth conditions are favorable for producers, but also for numerous competing bacteria. Given that these molecules possess excessively strong activity, bacteria reduce their production by silencing *lcnB* transcription several-fold. On the other hand, when bacteria enter a peptide-poor environment, PrtP is highly expressed on the cell surface, as a tool for obtaining small peptides from proteins. Consequent digestion of present LcnB molecules renders them less active but leads to initial elevation of small peptides or free amino acids in the environment. As a consequence of lower LcnB activity, *lcnB* transcription magnifies.

It should be mentioned that in *L. lactis* BGMN1-501, genes *lcnB* and *prtP* are located on the same genetic element. It is tempting to assume that these genes co-evolved, at least in some lactococci. On the other hand, there are a number of strains harboring only one of these genes, as well as those which harbor them on separate plasmids (Miladinov et al., 2001). Hence, we must not exclude the possibility of a non-functional adverse effect of proteinase on bacteriocin and that there is another explanation of bacteriocin activity enhancement parallel to attenuation of its gene expression, but we consider this not accidental. It should be noted that PrtP proteinase also digests itself in a process of autocatalytic release from cells into the medium (Laan and Konings, 1989; Bruinenberg et al., 1994; Flambard and Juillard, 2000). It is also known that bacteria use proteinases as a defense mechanism against attacks by bacteriocin-producing bacteria (Nawrocki et al., 2014), but it is still debatable why bacteria would impair their own bacteriocin. The fact that it does not destroy it completely implies some regulation-based explanation. It is certain that as a consequence of this action a pool of small peptides rapidly generates in the close vicinity of nitrogen-depleted bacterial cells. Perhaps this “self-digestion” has a physiological role in some sort of first aid for bacteria which find themselves in a peptide-poor environment, especially if we take into account that antimicrobial activity for inhibiting the growth of neighboring bacteria is retained.

Defining the exact peptide bond or bonds that are cleaved in LcnB by the proteinase will lead to a better understanding of the structure-function relationships in the bacteriocin and help define its receptor binding domain as well as its mode of action.

In conclusion we wish to point out that our results are of both fundamental and applicable significance. They provide new insight into bacteriocin activity, opening a new field of transcriptional and translational regulation of bacteriocin expression. Also, it is reasonable to consider that similar regulation may exist in bacteriocins that are already used in food preservation or in ones that might be used in the future. Since it is not unusual for bacteriocin and proteinase producers to be applied in food production, our work indicates that their interaction should be taken into account when planning the final concentration of desired active molecules.

## AUTHOR CONTRIBUTIONS

GV, NM, and MM contributed to acquisition and analysis of data; BJ, IS, ZR, and DF contributed to design of the work, analysis, and interpretation of the data; GV, NM, and BJ drafted the manuscript; MK contributed to the conception of the work and did final approval of the version to be published.

## Conflict of Interest Statement

The authors declare that the research was conducted in the absence of any commercial or financial relationships that could be construed as a potential conflict of interest.
